# Spatial Distribution Dynamics of Sensory Disturbances in the Treatment of Obesity-Related Meralgia Paresthetica Using Transcutaneous Electrical Nerve Stimulation

**DOI:** 10.3390/jcm14020390

**Published:** 2025-01-09

**Authors:** Mustafa Al-Zamil, Natalia G. Kulikova, Natalia A. Shnayder, Natalia B. Korchazhkina, Marina M. Petrova, Numman Mansur, Larisa V. Smekalkina, Zarina M. Babochkina, Ekaterina S. Vasilyeva, Ivan V. Zhhelambekov

**Affiliations:** 1 Department of Physiotherapy, Faculty of Continuing Medical Education, Peoples’ Friendship University of Russia, 117198 Moscow, Russia; kulikovang777@mail.ru; 2 Department of Restorative Medicine and Neurorehabilitation, Medical Dental Institute, 127253 Moscow, Russia; zzangieva2008@yandex.ru (Z.M.B.); aiybolit69@mail.ru (I.V.Z.); 3 Department of Sports Medicine and Medical Rehabilitation, I.M. Sechenov First Moscow State Medical University, 119991 Moscow, Russia; smekalkinal@bk.ru; 4 Institute of Personalized Psychiatry and Neurology, V.M. Bekhterev National Medical Research Centre for Psychiatry and Neurology, 192019 Saint Petersburg, Russia; 5 Shared Core Facilities “Molecular and Cell Technologies”, Professor V. F. Voino-Yasenetsky Krasnoyarsk State Medical University, 660022 Krasnoyarsk, Russia; stk99@yandex.ru; 6 Department of Restorative Medicine and Biomedical Technologies, Federal State Educational Institution of Higher Education, Moscow State University of Medicine and Dentistry Named After A.I. Evdokimov, Ministry of Health of Russia, 127473 Moscow, Russia; n9857678103@gmail.com (N.B.K.); e_vasilieva@inbox.ru (E.S.V.); 7 City Clinical Hospital Named After V.V. Vinogradov, 117292 Moscow, Russia; d-64-158@mail.ru

**Keywords:** meralgia paresthetica, high-frequency low amplitude TENS, low-frequency-high amplitude TENS, pain drawing, body drawing, McGill pain questionnaire, lateral femoral cutaneous nerve, ASIS, gate control theory of pain

## Abstract

**Background:** To date, there have been no studies on the dynamics of areas of pain, paraesthesia and hypoesthesia after the use of various transcutaneous electrical nerve stimulation in the treatment of meralgia paresthetica. **Methods:** In this pilot study, we observed 68 patients with obesity-related bilateral meralgia paresthetica. Pain syndrome, paraesthesia symptoms, and hypoesthesia were evaluated using 10-point scores. In addition, pain drawing (PD) was used to determine the area of the spatial distribution of pain syndrome and paraesthesia symptoms, and body drawing was used to determine the area of hypoesthesia. Sham TENS was performed in the control group, and effective TENS was performed in the treatment group. The treatment group consisted of two subgroups. One subgroup underwent HF-LA TENS, and the second subgroup underwent LF-HA TENS. **Results:** Despite the greatest analgesic effect observed from HF-LA TENS, which was assessed using scoring methods, during and after treatment, the reduction in the area of pain and paraesthesia symptoms and the area of hypoesthesia was moderate, short-term, and reversible. In contrast, LF-HA TENS had a pronounced analgesic and sustained anti-paraesthesia effect, manifested by a noticeable decrease in pain and paraesthesia symptoms area in PD, gradually increasing during the first 2 months of follow-up and accompanied by an irreversible prolonged decrease in the area of hypoesthesia. **Conclusion:** The areas of paraesthesia and hypoesthesia correlate with affective reactions to long-term chronic pain, which noticeably regress under the influence of LF-HA TENS compared to HF-LA TENS.

## 1. Introduction

Meralgia paresthetica (MP) is one of the most common neuropathies of the lower extremities, associated with compression of the lateral femoral cutaneous nerve (LFCN), clinically manifested by pain, paraesthesia, and sensory loss in the anterolateral thigh. MP is also known as Bernhardt–Roth syndrome, lateral femoral cutaneous nerve syndrome, or lateral femoral cutaneous neuralgia [1,2,3]. German neurologist Bernhardt described the symptoms of this disease in detail in 1895, and a few days later, Russian neurologist Roth published an article in which he coined the term “meralgia” from the Greek words meros, meaning hip, and algos, meaning pain, creating the term meralgia paresthetica, which describes the nature of this disease in two words [4]. However, the first description of this disease was given 10 years earlier by the German surgeon Werner Hager in 1885 as a “traumatic neuritis” of the lateral cutaneous nerve of the thigh [5,6].

LFCN is formed by the higher lumbar spinal nerves L2 and L3. The nerve fibers originate from the lumbar plexus in the posterior abdomen, emerge from the lateral border of the psoas major muscle, pass obliquely down the anterior surface of the ilium, and pass to the thigh between the anterior superior iliac spine (ASIS) and the inguinal ligament [7]. Nerve damage can occur anywhere during the passage of the nerve. However, the most common location is the narrow distance between the ASIS and the inguinal ligament [8].

Despite the absence of motor deficits and gait disturbances, many patients with MP experience disability and decreased quality of life due to limitation of motor activity in order to minimize pain and paraesthesia symptoms [9]. The relevance of this disease is growing every year as the prevalence of obesity and diabetes mellitus increases, which leads to an increase in the incidence of MP to 32–64 new cases per 100,000 people per year [10,11]. Obesity doubles the risk of developing MP due to abdominal protrusion and increased pressure on the inguinal ligament, causing it to move upward and inward, narrowing the distance between the ASIS and the inguinal ligament for the passage of LFCN [12].

In clinical practice, the diagnosis of MP is made in the presence of clinical symptoms and the identification of sensory symptoms characteristic of this disease during physical examination [13]. Sensory nerve conduction studies and somatosensory evoked potentials can be used to confirm the diagnosis and clarify the location and nature of nerve damage. However, due to technical complexity, the results of neurophysiological studies are not always conclusive, which increases their technical limitations and significantly reduces their specificity and sensitivity [4]. Ultrasound is a useful modality for LFCN assessment in clinically suspected MP and is more sensitive to abnormalities than MRI [13,14].

LFCN impairment is characterized by hypoesthesia and paraesthesia in an irregular oval shape in the anterolateral part of the thigh with a gradual loss of sensation from the center of the oval to the periphery [15,16]. The severity of hypoesthesia in MP correlates with an increase in the area of the hypoesthesia oval and the area of maximal sensation loss in its central part [1,15,17]. Unpleasant and, in rare cases, unbearable paraesthesia symptoms can spread further from the boundaries of the hypoesthesia oval, which complicates the diagnosis of this disease, and in many cases, the symptoms of the disease can be mistaken for low back pain [18,19].

Despite the high efficiency of surgical management of MP in many cases, conservative treatment remains the method of choice and the first approach in the treatment of most patients with this disease [20,21]. The conservative management of MP includes patient education about avoiding tight clothing, weight loss, and pharmacotherapy with the use of non-steroidal anti-inflammatory drugs (NSAIDs) and central analgesics [1]. Indeed, in many cases, replacing belts with suspenders in patients with obesity can cause spontaneous pain relief and a detectable sensory recovery [17]. However, in most cases, the use of pharmacotherapy alone does not sufficiently reduce paraesthesia and hypoesthesia [15]. However, in many studies, an additional combination of physiotherapeutic methods can significantly increase the effectiveness of pharmacotherapy in the treatment of MP [22,23,24,25].

Transcutaneous electrical nerve stimulation (TENS) is a non-invasive, non-pharmacological, and simple intervention that has a pronounced analgesic effect. The analgesic effect of TENS is based on many factors. The release of endogenous endorphins is one of the crucial analgesic factors of TENS [26]. Other neurogenic mechanisms of analgesia involve the activation of opioid-mediated antinociception in many central structures (descending inhibitory pathway), including the rostroventromedial medulla and periaqueductal gray [27,28,29]. Additionally, local anti-inflammatory, antinociceptive effects, improved microcirculation, and accumulation of growth factor lead to a noticeable decrease in peripheral sensitization. However, all these peripheral and central mechanisms play an important role in reducing central sensitization, which plays a crucial role in prolonging the analgesic effect of TENS [30].

The recovery effect of TENS in the treatment of neuropathies has been demonstrated in many experimental [31,32,33] and clinical studies [34,35,36,37,38]. The results of treatment were not limited to a decrease in sensory and motor deficits but extended to regression of electromyography changes in the affected nerves [39,40]. Several studies have shown the important role of TENS in accelerating regenerative processes in damaged peripheral nerves [41] and denervated muscles [42,43], as well as locally improving vascularization and the antinociceptive and anti-inflammatory responses in the stimulation zone [44,45].

Many studies have been reported in the literature demonstrating the high effectiveness of TENS in the treatment of MP [4,10,15,17,46]. However, a comparative analysis of high-frequency low-amplitude TENS (HF-LA TENS) and low-frequency high-amplitude TENS (LF-HA TENS) in the treatment of MP was not carried out. In addition, the dynamics of the areas of distribution of hypoesthesia and paraesthesia after the use of various TENS modalities have not yet been studied.

The aim of our research is to compare the efficiency of HF-LA TENS and LF-HA TENS in resolving the spatial distribution of pain, symptoms of paresthesia, and hypoesthesia in the treatment of patients with meralgia paresthetica associated with obesity immediately after treatment and in the long term.

## 2. Materials and Methods

### 2.1. Study Design and Population

In this pilot study, we observed 68 patients with meralgia paresthetica and bilateral lower limbs; ICD-10-CM code: G57.13 [47].

Inclusion criteria:

1. European.
2. Men and women over 25 years old and up to 60 years old.
3. All patients have obesity, with a BMI ranging from 30.0 to 39.0 kg/m^2^.
4. A duration of pain syndrome from 3 months to 2 years.
5. The pain syndrome is localized maximally in the anterolateral thigh.
6. Pain on the VAS scale—5 points or higher.
7. Decreased SNAP amplitude below 3 μV on LFCN electromyography or unevoked SNAP.
8. Signed an agreement to participate in this study.

Exclusion criteria:

1. History of the allergic effect of any of the drugs used;
2. Cognitive and mental disorders;
3. Diabetes mellitus;
4. The presence of cardiac arrhythmias or hemodynamic decompensation;
5. Implanted electronic devices;
6. Pregnancy;
7. Other courses of physiotherapy or acupuncture are carried out in parallel.

All patients gave written consent to participate in the study and clinical treatment after explaining their health status, the purpose and benefits of diagnostic tests, treatment procedures, and possible complications or adverse events during the examination and treatment period.

Our research was approved by the local medical ethical committee of Medical Dental Institute, Moscow, Russia (protocol No. 11, 5 April 2022). Government registration number: 0120.0807480- Higher Attestation Commission of the Russian Federation. International registration number: ISRCTN47534508—ISRCTN registry. All examinations and treatment procedures were conducted according to the 1984 Declaration of Helsinki and its subsequent amendments. All patients consented to the publication of the manuscript after reading it in its entirety, including text and figures.

There was no compensation to participants or researchers for participating in this study. The research was conducted within the research program of the Department of Restorative Medicine and Neurorehabilitation, Medical Dental Institute, Moscow, Russia.

In this study, 122 patients with bilateral meralgia paresthetica were evaluated for eligibility (110 women, 112 men). Overall, 54 of these patients were excluded, 42 of which did not meet the inclusion criteria, and 12 patients decided not to continue their participation. A total of 68 patients (female—35, male—33) met all inclusion criteria and were distributed in a 1:1:1 ratio. Two patients were excluded from the study due to loss of follow-up.

**Figure 1:**
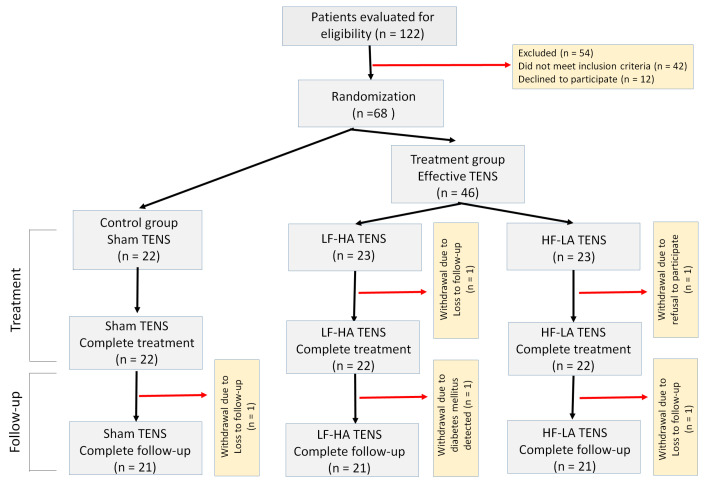
Flow chart of study population selection. Note: TENS—transcutaneous electrical nerve stimulation; LF-HA—low-frequency high-amplitude; HF-LA—high-frequency low-amplitude.

Ultimately, the number of patients who could complete treatment was reduced to 66 (female—34, male—32). All patients underwent standard pharmacotherapy. In addition, the control group (*n* = 22) received a course of sham TENS. Contrastingly, the treatment group (*n* = 44) passed a course of effective TENS and was divided into two subgroups. In the 1st subgroup, HF-LA TENS (*n* = 22) was performed, and in the 2nd subgroup, LF-HA TENS was performed (*n* = 22). Only 63 patients completed the 4-month follow-up. Three patients were withdrawn from the research after they were diagnosed with diabetes mellitus and lost to follow-up (Figure 1).

Demographic and clinical characteristics of the studied patients are demonstrated in Table 1. The study participants ranged in age from 28 to 59 years and averaged 46.7 ± 2.85 years of age. In the control group and treatment subgroups, the ratio of male to female study participants was 1:1. BMI in all patients was higher than 30 kg/m^2^ but less than 40 kg/m^2^, and the average value was 34.7 ± 2.50 kg/m^2^. The duration of MP symptoms lasted from 5 to 20 months and averaged 15.9 ± 3.24 months. The severity of pain in the anterolateral thigh was assessed on a VAS from 6.0 to 10.0 points; the average result was 7.82 ± 1.20 points. No reliable differences in the number of patients, average age, sex ratio, BMI, duration of the disease, and severity of pain were detected between groups.

### 2.2. Sample Size Calculation

The smallest number of participants in each group was estimated using the sample size calculator at https://clincalc.com/stats/samplesize.aspx [48]. A previous study compared the effectiveness of TENS with sham TENS in the treatment of MP [49]. The severity of pain on the VAS scale (M ± SD) significantly decreased after the use of effective TENS from 7.4 ± 2.3 points to 3.3 ± 1.5 points. The detected analgesic effect was lower than after sham TENS by 41.0%. Using these data, the minimum number of patients in each group was calculated according to the following parameters: power value = 95%, probability of type I error = 0.01, and expected level of significance (*p*-value) = 0.05. Consequently, the sample size was finally determined to be 15.

### 2.3. Clinical Examinations and Diagnostics

Neurological clinical examination was performed by a board-certified neurologist blinded to participant status. During the neurological examination, the identified abnormalities were assessed in detail and recorded in special reports. Mental disorders, cognitive impairment, changes in cranial nerves, cerebellar and gait abnormality, motor deficits, reflex disorders, the development of pathological reflexes, and loss of sensation were studied and evaluated. To confirm the LFCN impairment in the ASIS projection, Tinel’s sign and pelvic compression test were examined. Tinel’s sign was considered positive if, in response to percussion or light tapping on the projection of the medial surface of the ASIS, paraesthesia developed in the form of tingling or a tingling sensation in the distal distribution of the injured nerve [50]. The pelvic compression test was considered positive if temporary alleviating of pain and paraesthesia occurred in response to downward hip force, resulting in relaxation of the inguinal ligament [20]. Anamnesis of the disease was collected, clarifying the presence of comorbidity, heredity, trauma, and intoxication, as well as clarifying the nature of the course of the identified symptoms in terms of frequency, duration, and severity.

#### 2.3.1. Pain Assessment

##### The Visual Analog Scale

The pain VAS has been widely used to assess pain worldwide since 1921 [51]. Patients independently indicate their subjective pain on an 11-point vertical or horizontal line, which corresponds to an 11-point scale from 0 (no pain) to 10 (unbearable pain). This method is used during neurological examinations in clinical practice and scientific studies, regardless of the nature of the pain syndrome, to evaluate the analgesic effect of many therapies in the treatment of many diseases [52]. VAS differs from descriptive ordinal scales pain assessment tools in its high sensitivity to small changes and simplicity for the patient and the doctor. In clinical research, pain numbering simplifies and makes comparative and correlation analyses more accurate.

##### McGill Pain Questionnaire

The McGill Pain Questionnaire (MPQ) was first proposed to assess various aspects of pain by Dr. Melzack of McGill University in Montreal, Canada, in 1975 [53]. This method was designed to assess the pain sensation, regardless of its severity and nature. Its peculiarity lies in the assessment of not only sensory pain signs, which may be the result of compression, irritation, damage, or inflammation but also affective reactions that develop as a result of the emotional system’s response to pain. To achieve this goal, the McGill Pain questionnaire consists primarily of three main classes of verbal descriptors: sensory, affective, and evaluative. Descriptors make it possible to characterize the pain sensation or reaction based on the subjective characteristics of the patient’s own pain experience. Descriptors are divided into subclasses. Each subclass consists of descriptors of one of the signs of pain in the sensory class and an emotional response to pain in the affective class. Each of the descriptors has a numerical value and minimum intensity, located first in order. The questionnaire was designed to provide quantitative measures of clinical pain that can be treated statistically. When completing the questionnaire, the patient can select one descriptor from each subclass, but not necessarily in each subclass. Two major measures can be derived from MPQ in statistical quantitative of clinical pain in sensory and affective classes: the pain rating index (PRI), based on the sum of numerical values of selected descriptors and the number of descriptors chosen. Evaluative classes can evaluate the present pain intensity based on a 1–5 verbal intensity scale [54]. In our study for comparative and correlation analyses, we used PRI of sensory and affective classes.

##### Pain Drawing

We conducted an assessment of pain distribution by PD based on the localization of pain using drawings on a body map to clarify the nature and pattern of pain and to display the topography of pain and paraesthesia by the patients themselves. Patients, at the request of physicians, draw the places where they felt pain and paraesthesia over the past 24 h [55]. PD is a highly informative method for the differential diagnosis of many pain conditions, which makes it possible to clarify the etiology of pain, the topographic localization of nerve damage, and the degree of nerve injury [56].

Many experimental and clinical studies have shown the convenience and reliability of PD in the assessment of severe and chronic pain conditions [57] and in predicting the outcome of analgesic treatment [58].

Recently, digital versions of PD have been developed using computer technology to transmit and record patient data [59]. However, drawings made with pen on paper are easier and more convenient for patients to record, but they are more complex and require more time for transmission, processing, and quantification of data by the researcher [60]. Nevertheless, this problem can be solved using specially designed software.

Localization of pain and paraesthesia is usually accomplished by circling the areas of the body where pain occurs, regardless of the magnitude of pain and paraesthesia in these areas [3]. Most often, the pattern of pain distribution in MP does not correlate with the severity of pain and paraesthesia assessed by VAS, and in many cases, the ratio of the pain and paraesthesia area to the entire anterolateral area of the thigh exceeds one. That is, pain and paraesthesia spread wider than the innervation zone of LFCN. According to many authors, the diffuse distribution of pain in PD and large areas of neuroanatomically illogical distribution are associated with the development of central sensitization [21] and affective reactions to chronic pain [15]. In MP, it is difficult to distinguish the area of pain from the area of paraesthesia in PD. Therefore, we decided to combine two PDs of pain and paraesthesia into one to localize the spread of pain and paraesthesia area (PPA) [16,17].

In our study, the self-reported PD was circled before treatment, immediately after treatment, as well as after 2 and 4 months of follow-up. All PD were downloaded to the computer by scanning. The area of the offended area, regardless of the intensity of the underline and fill, was calculated by adobe photoshop program CC 2019 (20.0.3) version. After determining PPA in square pixels, the resulting indicator is divided by the total anterolateral area of the thigh to quantify the PPA ratio. The total thigh area was determined on 2D lateral view images, using lines drawn from the intergluteal fold to the ASIS proximally and transverse knee axis distally.

#### 2.3.2. Tactile Sensation Assessment in Anterolateral Thigh

##### Rating of Paraesthesia in a 10-Point Scale

The patients themselves rated their unpleasant paraesthesia symptoms in the affected limbs using a 10-point visual analog scale, where 0 points corresponded to the absence of paraesthesia, and 10 points corresponded to an unbearable level of paraesthesia symptoms. Using a special questionnaire, patients assessed the intensity of paraesthesia over the past 24 h. Among the symptoms to evaluate paresthesia, we chose sensations such as burning, numbness, tingling, and electrical discharges localized in the affected area. Mean values were subtracted for each limb. Paresthesia was assessed before and after treatment, as well as 2 and 4 months after completion of treatment (follow-up period).

##### Rating of Hypoesthesia on a 10-Point Scale

The tactile sensation was assessed in dermatomes innervated by LFCN bilaterally. Tactile sensations on the anterolateral surface of the thigh were assessed in comparison with tactile sensations on the lower leg on a ten-point scale. Loss of sensation (anesthesia) corresponded to 10 points, and normal sensation (absence of hypoesthesia) corresponded to 0 points. In the test, for sensory evaluation, we used a Touch Test filament of 6.65 g.

##### Determination of the Area of Hypoesthesia

**Figure 2:**
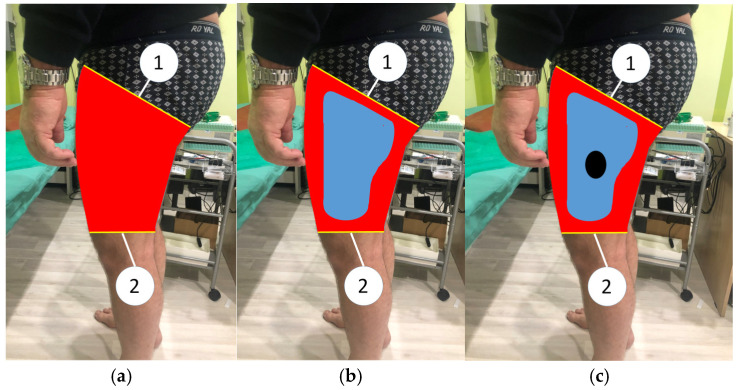
Evaluation of hypoesthesia in the left lateral femoral cutaneous nerve dermatome by calculating hypoesthesia ratio and maximum hypoesthesia ratio. Notes: (**a**) calculation of the total lateral thigh area (red color) between lines 1 and 2; (**b**) calculation of hypoesthesia ratio by dividing the area of hypoesthesia localized on neurological examination (blue color) by the total lateral thigh area. (**c**) Calculation of maximum hypoesthesia area by dividing the maximum hypoesthesia area localized on neurological examination (black color) by hypoesthesia area (blue color); 1—a line drawn from the intergluteal fold to anterior superior iliac spine; 2—the transverse axis of the knee.

The area of hypoesthesia is determined by pressing a TTF against the skin from the distal medial, lateral, superior, and inferior parts of the anterolateral thigh towards the dermatomes of LFCN. When patients notice a decrease in tactile sensations, they immediately report this to the researcher, who marks this point with a felt-tip pen. This procedure is repeated on each side. As a result, a line is drawn between the points, and an irregularly shaped oval is obtained. The second stage is to determine the zone of maximum hypoesthesia by pressing the TTF to the skin in the medial, lateral, superior, and inferior parts of the hypoesthesia zone towards the center of this zone. In all areas of examination, points are noted at which tactile sensation is significantly reduced to 3 points and below. As a result, all marked points are connected by a line and an uneven circle is obtained in the center of the hypoesthesia oval. The resulting boundaries of hypoesthesia on the lateral surface of the thigh were photographed and loaded into a computer for further processing in order to subtract the hypoesthesia ratio (H-ratio) and maximum hypoesthesia ratio (MH-ratio). The H-ratio is obtained by dividing the hypoesthesia area by the total lateral thigh area. The total lateral thigh area was determined on two-dimensional (2D) lateral views using lines drawn from the intergluteal fold to ASIS proximally and the transverse axis of the knee distally. MH-ratio is the result of dividing the maximum hypoesthesia area by the hypoesthesia area (Figure 2).

#### 2.3.3. Quality of Life Enjoyment and Satisfaction

Quality of Life Enjoyment and Satisfaction Questionnaire (Q-LES-Q-SF) is a highly sensitive method for assessing the level of pleasure and satisfaction experienced by patients during the last week [61]. Evaluation of comprehensive enjoyment and satisfaction was carried out using scores on physical condition, emotional state, profession, family atmosphere, social and family interrelation, daily activity, sex drive, financial situation, self-confidence, welfare, satisfaction with treatment, and life. The Q-LES-Q-SF is a self-report questionnaire consisting of 16 items on a 5-point scale. The minimum total score is 16, and the maximum is 80. The total score is expressed as a percentage of the maximum total score for all items (0–100). The higher the scores, the higher the quality of life, pleasure, and satisfaction. Many studies have found that scores below 70% are often associated with decreased quality of life [62].

#### 2.3.4. Laboratory Examination

Laboratory examination of complete blood count with erythrocyte sedimentation rate was determined. Additionally, rheumatoid factor, IgM, Anti-cyclic citrullinated peptide antibodies, C-reactive protein, antinuclear antibodies, anti-double-stranded DNA, complement C3 and C4, serum uric acid, serum Folate, vitamin B12, 25-hydroxy vitamin D, HbA1, and creatine kinase were assessed.

#### 2.3.5. Electroneuromyography

Nerve conduction velocity (NCV) and sensory nerve action potential (SNAP) amplitude of the LFCN were recorded bilaterally using ENMG. The antidromic stimulation method was used. SNAP of the LFCN was detected using distal surface electrodes located approximately 30 cm caudal to the ASIS. Electrical stimulation was carried out at the ASIS level [63,64]. ENMG was performed before treatment and 2 months after treatment.

#### 2.3.6. Magnetic Resonance Imaging Founds

Magnetic resonance examination (MRI) was carried out on scanners of at least 1.5 Tesla. MRI was performed to exclude lumbar disk herniation, possible compression of nerves at the spinal level, degenerative conditions of the sacroiliac and hip joints, and bone pathologies of the spine and pelvis associated with dysmorphism, oncology, and osteomyelitis.

#### 2.3.7. Ultrasonography

Each patient was positioned supine and was scanned bilaterally by an investigator with 10 years of experience using a Mindray C18-5 (Shenzhen, China) ultrasound scanner with an 18 MHz linear array transducer. LFCN was identified in ASIS. Normally, the LFCN can be easily identified and usually has an ovoid hypoechoic structure with hyperechoic dots within it. The cross-sectional area of the nerve at this location was measured bilaterally. The distance between LFCN and ASIS was also measured. By observing the course of the nerve, the cable-like structure, vascularization, and other morphological changes in the nerve were studied [13]. Ultrasonography (US) was performed before treatment and 2 months after treatment.

### 2.4. Treatment

#### 2.4.1. Pharmacotherapy

Pharmacotherapy included etoricoxib 90 mg for 10 days, vitamin B1, B6, and B12 complex for 5 days (intramuscularly), and Voltaren gel 2 times a day for 15 days in combination with gabapentin at a dose of 300 mg 3 times a day for 3 months.

#### 2.4.2. Transcutaneous Electrical Nerve Stimulation

**Figure 3:**
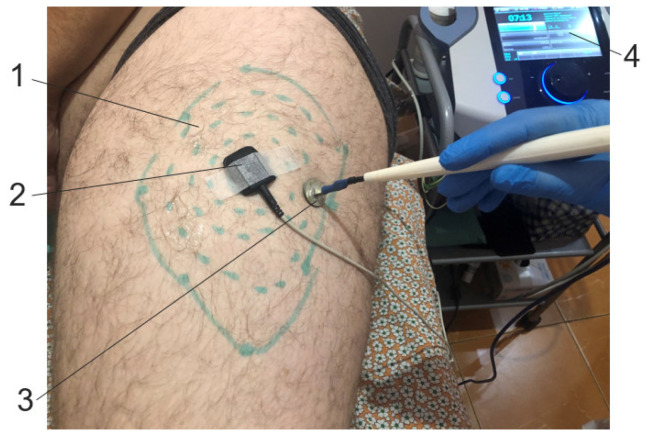
TENS technique of the lateral femoral cutaneous nerve. Notes: 1: hypoesthesia area; 2: anode; 3: cathode pen electrode; 4: electrical stimulation device.

TENS was carried out with rectangular monopolar electrical pulses. A 1 cm^2^ anode was fixed in the center of the hypoesthetic oval, identified during the clinical examination of the anterolateral thigh. The cathode pen electrode of 1 cm^2^ was not fixed and moved around the anode from the center laterally in a spiral. Stimulation was applied in the hypoesthesia area. The distance between stimulation points was 1–2 cm, and the duration of stimulation at each point was 10 s. Stimulation was carried out for 15 min on each side. The total time for each procedure was 30 min. A total of 15 procedures were performed every other day for a month (Figure 3).

Our study used three types of TENS: Sham TENS, LF-HA TENS, and HF-LA TENS. The characteristics of the applied current are shown in Table 2.

Electrical impulses were generated by a BTL-4000 smart/premium device (BTL Industries Ltd., Hertfordshire, UK) with registration number RAN 2020/12648, dated 24 November 2020.

## 3. Results

### 3.1. Clinical Examination

In this study, we included only patients with clinical symptoms of MP developing bilaterally against a background of obesity. Examination of mental status or cognitive function revealed no abnormalities in all patients. Eye movements were within normal limits without nystagmus. No voice changes or swallowing disorders were detected. No motor deficit was detected. Bladder and bowel dysfunction were not diagnosed. No pathological reflexes were recorded. No gait or coordination disorders were detected. No bilateral or unilateral root innervation disorders were found. Signs of neurogenic or vascular claudication were absent. Nevertheless, neurological examination revealed other clinical disorders, such as pain, hypoesthesia, and paraesthesia in the dermatomes of LFCN bilaterally. Moreover, in all patients examined, one or both Tinel’s symptoms and the pelvic compression test were positive to varying degrees of severity. None of the patients experienced side effects after using pharmacotherapy or TENS.

### 3.2. Pain Syndrome

#### 3.2.1. Visual Analog Scale

The initial level of pain syndrome on the VAS scale was high in the control group and treatment subgroups and averaged 6.45 ± 0.14 points. The mean values in the study groups did not differ significantly from each other.

After treatment, pain regression was significant in all groups and averaged 19.7% in the sham TENS group (t = 4.41, *p* = 0.0001), 46.3% in the LF-HA TENS group (t = 14.5, *p* = 0.0001), and 75.4% in HF-LA TENS group (t = 23.3, *p* = 0.0001).

During the first 2 months of follow-up, the analgesic effect in the sham TENS group remained without significant changes (t = 0.35, *p* = 0.72). However, opposite significant changes were found in patients after LF-HA TENS by 28.6% (t = 6.85, *p* = 0.0001) and after HF-LA TENS by −125% (t = 10.4, *p* = 0.0001). Despite the pronounced negative dynamics in the HF-LA TENS group, the pain level was lower than in the sham TENS group by 28% (t = 5.49, *p* = 0.0001) but higher than in the LF-HA TENS group by 44% (t = 5.18, *p* = 0.0001).

**Figure 4:**
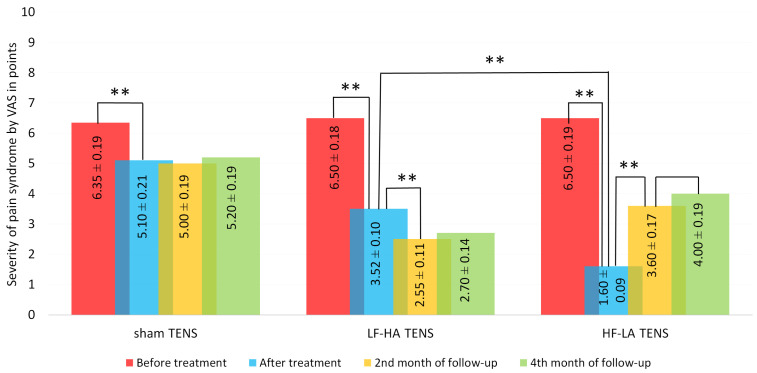
Dynamics of pain syndrome after treatment and in the follow-up period in TENS groups. Notes: VAS—visual analog scale; TENS—transcutaneous electrical nerve stimulation; LF-HA—low-frequency high amplitude; HF-LA—high-frequency low-amplitude; #—*p* > 1; **—*p* ≤ 0.01.

At the end of the 4th month of follow-up, there were no noticeable changes in pain intensity compared to the recorded results in the 2nd month of observation. As a result, effective TENS had a greater analgesic effect than sham TENS by 1.68 times (t = 6.88, *p* = 0.0001), and LF-HA TENS turned out to be more effective than HF-LA TENS by 52% (t = 5.49, *p* = 0.0001) (Figure 4).

#### 3.2.2. The McGill Pain Questionnaire

Before treatment, sensory and affective dimensions of pain significantly did not differ between TENS groups and averaged 15.2 ± 0.35 scores in the sensory class and 8.23 ± 0.21 scores in the affective class.

After treatment, the reduction in sensory dimensions of pain was, on average, 13.2% (t = 3.20, *p* = 0.003) in the sham TENS group and 54.0% (t = 13.2, *p* = 0.0001) in the effective TENS group. Whereas significant changes in affective dimensions of pain were registered only after effective TENS and averaged 47.1% (t = 6.16, *p* = 0.0001). A comparison of the results obtained after the use of different modalities of effective TENS revealed that the reduction in sensory class was superior after HF-LA TENS by 30.9% (t = 5.66, *p* = 0.0001), and in affective class after LF-HA TENS by 79.3% (t = 8.78, *p* = 0.0001).

At the end of the 2nd month of follow-up, no changes were noted in sensory and affective classes after sham TENS. In the LF-HA TENS group, the significant reduction in sensory and affective classes continued to 32.9% (t = 5.78, *p* = 0.0001) and 25% (t = 4,03, *p* = 0.0002), respectively. In contrast, the HF-LA group experienced significant pain relapse in the sensory and affective classes at 35.6% (t = 5.17, *p* = 0.0001) and 18.2% (t = 4.02, *p* = 0.0002), respectively.

A 4-month follow-up showed that effective TENS had a prolonged analgesic effect that was higher than sham TENS by 47.0% (t = 11.5, *p* = 0.0001) in the sensory class and 46.5% (t = 8.83, *p* = 0.0001) in the affective class. Compared to the baseline pain level, the analgesic effect was maintained at 53.9% (t = 9.17, *p* = 0.0001) in the sensory class and 44.5% (t = 7.90, *p* = 0.0001) in the affective class. The analgesic effect of LF-HA TENS compared to HF-LA TENS was higher in the sensory class by 29.3% (t = 5.06, *p* = 0.0001) and in the affective class by 57.8% (t = 9.20, *p* = 0.0001) (Table 3).

#### 3.2.3. Pain Drawing

Before treatment, in PD, it was revealed that pain and paraesthesia (tingling, pricking, chilling, burning, numbness) were asymmetric in 81% (*n* = 51) of patients, with a predominance of the right side in 65% (*n* = 65) of them. In 83% (*n* = 106) of the limbs, pain and paraesthesia were localized within the LFCN dermatome. However, in 20 limbs, the localization of pain and paraesthesia was noted as being proximal to a line drawn from the intergluteal fold to the anterior superior iliac spine and distal to the transverse axis of the knee joint. As a result, the PPA ratio in many of these cases turned out to be greater than one. Overall, pretreatment PPA ratios were greater than 50% in most limbs and averaged 74.6 ± 5.9%, with no significant differences between TENS groups.

After treatment, a significant reduction in the PPA ratio was recorded after effective TENS by 44.3% (t = 3.48, *p* = 0.0012) but not after sham TENS. Notably, the reduction in the PPA ratio after LF-HA TENS was greater than that after HF-LA TENS by 58.4% (t = 2.92, *p* = 0.006).

At the end of the 2nd month of follow-up, there was a significant decrease in the PPA ratio only in the LF-HA TENS group by 28.8% (t = 2.02, *p* = 0.045). However, it is important to underline that the PPA ratio after HF-LA TENS remained without negative dynamics.

**Figure 5:**
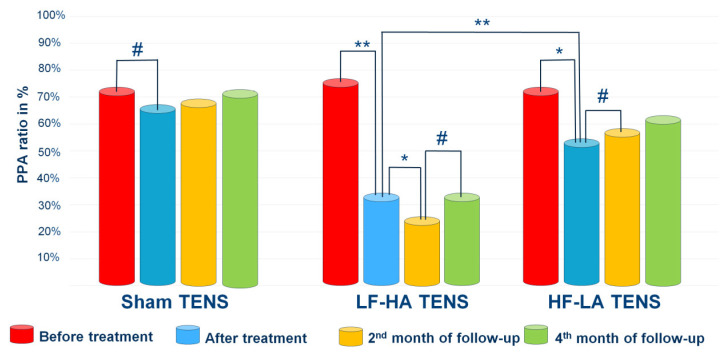
Dynamics of PPA ratio after treatment and in the follow-up in control group and treatment subgroups. Notes: TENS—transcutaneous electrical nerve stimulation; LF-HA—low-frequency high-amplitude; HF-LA—high-frequency low-amplitude; PPA ratio = pain and paraesthesia area ratio; #—*p* > 1; *—*p* ≤ 0.05, **—*p* ≤ 0.01.

At the end of 4th month of follow-up, there were no significant changes compared with the results obtained after treatment or with the results obtained after 2 months of follow-up in all TENS groups. Nevertheless, according to the data presented in Figure 5, a significant long-term decrease in the PPA ratio relative to baseline values was observed after LF-HA TENS by 57% (t = 4.90, *p* = 0.001). At the same time, the 15.8 4% (t = 1.36, *p* = 0.175) decrease in the PPA ratio after HF-LA TENS was not statistically significant.

**Figure 6:**
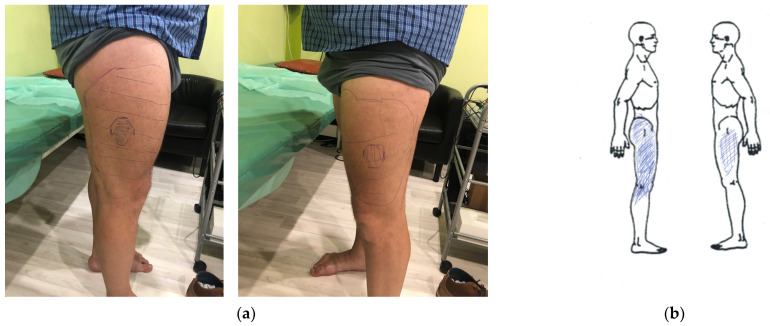
Areas of hypoesthesia and paraesthesia in a patient with bilateral meralgia paresthetica. (**a**) The zone of hypoesthesia (indicated by an oval line) and maximum hypoesthesia (shaded circle in the center); (**b**) the zone of paraesthesia (shaded with blue lines).

Our results show that pain and paraesthesia do not always correspond to the zone of hypoesthesia. In one and the same patient, almost identical zones of hypoesthesia have different degrees of severity and areas of pain and paraesthesia (Figure 6). This is due to the acute development of symptoms on one side, accompanied by severe pain and paraesthesia, which become much larger and more widespread than on the other side, with a zone of hypoesthesia already formed in the non-acute period.

### 3.3. Rating of Paraesthesia in a 10-Point Scale

**Figure 7:**
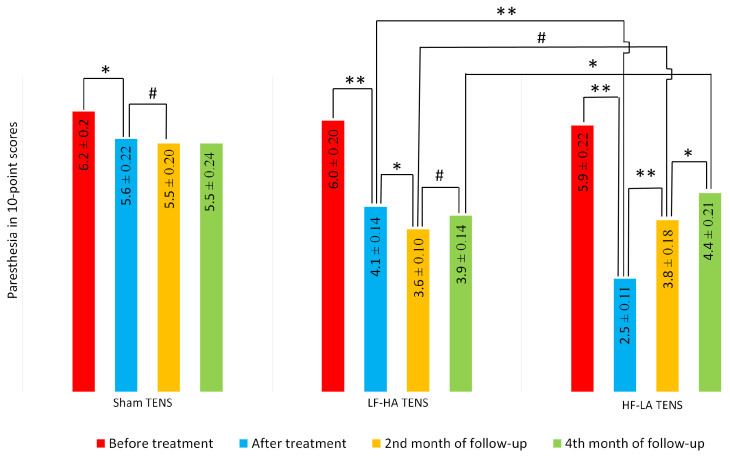
The dynamics of paraesthesia after treatment and in the follow-up in the control group and treatment subgroup. Notes: TENS—transcutaneous electrical nerve stimulation; LF-HA—low-frequency high-amplitude; HF-LA—high-frequency low-amplitude; PPA ratio = pain and paraesthesia area ratio; #—*p* > 1; *—*p* ≤ 0.05, **—*p* ≤ 0.01.

Most patients noted the presence of burning, numbness, tingling and electrical charge sensations in each of the affected limbs of varying degrees of severity. For example, one of the patients rated the severity of burning as 10 points, numbness as 4 points, tingling as 5 points, and electric charge as 3 points. Indeed, the varying severity of paraesthesia symptoms complicated the comparison process, and therefore, in line with our previous studies, we decided that the average of the four most common symptoms would be most reliable for comparative analysis between groups (Figure 7).

Before treatment, the mean severity of paraesthesia symptoms varied between 5 and 9 scores and averaged 6.03 ± 0.18. In all study groups, paraesthesia symptoms were significantly similar in nature and severity.

After treatment, the decrease in severity of paraesthesia symptoms was registered in all TENS groups by 9.7% (t = 2.12, *p* = 0.036) after sham TENS, by 31.7% (t = 7.60, *p* = 0.001) after LF-HA TENS and by 57.6% (t = 13.8, *p* = 0.0001) after HF-LA TENS. Our analysis revealed that the effect of effective TENS is 3.6 times greater than the effect of sham TENS. A comparative study of HF-LA TENS and LF-HA TENS was parallel to the dynamics of pain according to VAS, as a result of which HF-LA TENS was superior in the relief of paraesthesia by 82% (t = 9.82, *p* = 0.001).

At the end of the 2nd month of follow-up, the symptoms of paraesthesia significantly decreased in the LF-HF TENS group by 12.2% (t = 2.9, *p* = 0.006) and, conversely, increased in the HF-LF TENS group by 52.0% (t = 6.16, *p* = 0.001). There were no significant differences in results between the two groups.

At the end of 4 months of observation, our analysis revealed a prolonged effect of LF-HA TENS and HF-LA TENS, which was manifested by a regression of the level of paraesthesia by 35.0% (t = 8.60, *p* = 0.0001) and by 25.4% (t = 4.90, *p* = 0.001), respectively, compared to the baseline. It is worth noting that the reduction in paraesthesia symptoms after 4 months of sham stimulation averaged 11.3% (t = 2.24, *p* = 0.03).

### 3.4. Assessment of Impaired Tactile Sensation in Anterolateral Thigh

#### 3.4.1. Rating of the Hypoesthesia Severity on a 10-Point Scale

**Figure 8:**
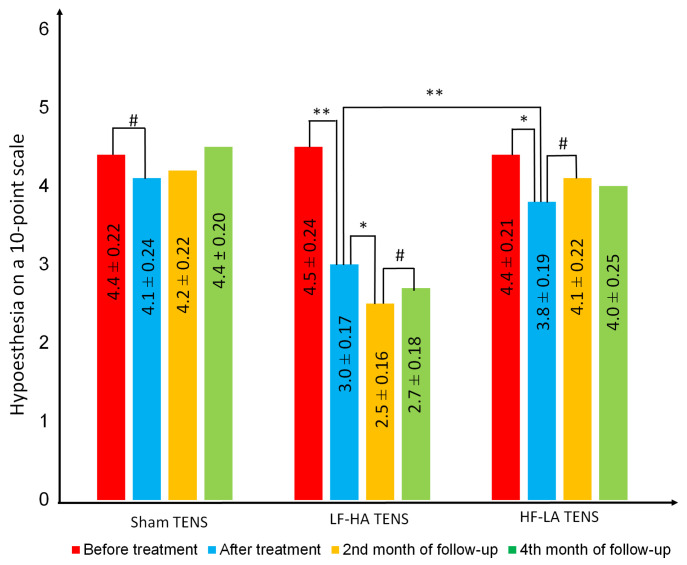
The dynamics of hypoesthesia after treatment and in the following control group and treatment subgroups. Notes: TENS—transcutaneous electrical nerve stimulation; LF-HA—low-frequency high-amplitude; HF-LA—high-frequency low-amplitude; #—*p* > 1; *—*p* ≤ 0.05, **—*p* ≤ 0.01.

Based on the results of assessing the symptoms of paraesthesia and pain before treatment, the severity of hypoesthesia was significantly lower by 28.3% and averaged 4.43 ± 0.18 points. Hypoesthesia was approximately at the same level in all TENS groups (Figure 8).

After treatment, a decrease in the severity of hypoesthesia was registered only in the effective TENS groups and averaged 23.8% (t = 4.7, *p* = 0.001), while LF-HA TENS was 1.4 times superior in recovery of tactile sensation (t = 8.25, *p* = 0.0001).

At the end of the second month of follow-up, a moderate fall in hypoesthesia was recorded only in the LF-HA TENS group. As a result, the level of hypoesthesia in this group after treatment continued to decrease by another 17.7% (t = 2.14, *p* = 0.038).

Observation for 4 months showed that in the long-term period, the decrease in hypoesthesia was persistent in all groups. However, minor and non-significant changes in the HF-LA TENS group at the end of the 2nd and 4th months of follow-up made the difference between the values at the end of follow-up and before treatment statistically insignificant. As a result, hypoesthesia was less than the initial level by 40% (t = 6.00, *p* = 0.0001) after LF-HA TENS and by 9.90% (t = 1.41, 0.165) after HF-LA TENS.

#### 3.4.2. Determination of the Area of Hypoesthesia

**Figure 9:**
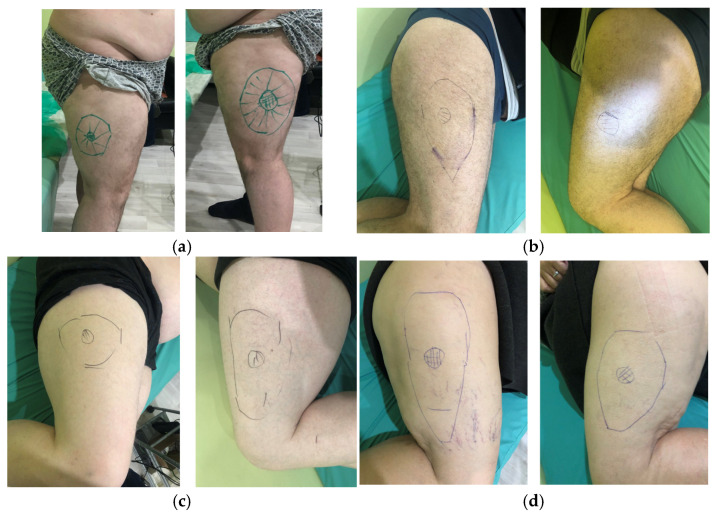
The zone of hypoesthesia (indicated by an oval line) and maximum hypoesthesia (shaded circle in the center) in 4 cases. (**a**) 45-year-old man, (**b**) 52-year-old man, (**c**) 57-year-old man, (**d**) 63-year-old woman.

Despite the high degree of paraesthesia and pain in severity and distribution in the anterolateral thigh area, the area of detected hypoesthesia in many cases was not correspondingly large (Figure 9). In all patients, the hypoesthesia zone had an irregular oval shape. In all patients, hypoesthesia progressed gradually, starting from the central parts of the LFCN dermatome with uniform distribution to distant sides. In our study, we were the first to use a new method for assessing hypoesthesia by determining the area of the central zone of pronounced hypoesthesia. For this, we calculated the MH-ratio, which was strongly correlated with the H-ratio, disease duration, and BMI and weakly correlated with the severity of pain and paraesthesia, and the PPA-ratio.

Before treatment, the h-ratio was in the range of 10–80% and averaged 42.9 ± 3.55%. No statistically significant differences were found between TENS groups. No crucial differences were found in the mean of the H-ratio and MH ratio between the right and left sides. However, the area of hypoesthesia was unsymmetrical in 83% (*n* = 55) of patients (Figure 6).

**Figure 10:**
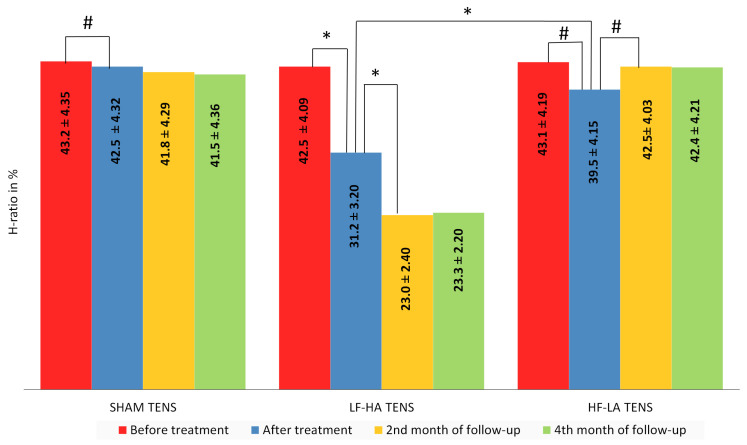
The dynamics of the hypoesthesia area in the anterolateral thigh after treatment and in the follow-up in the control group and treatment subgroups. Notes: H-ratio—an area of hypoesthesia divided by the anterolateral area of the thigh, in %;TENS—transcutaneous electrical nerve stimulation; LF-HA—low-frequency high-amplitude; HF-LA—high-frequency low-amplitude; #—*p* > 1; *—*p* ≤ 0.05.

**Figure 11:**
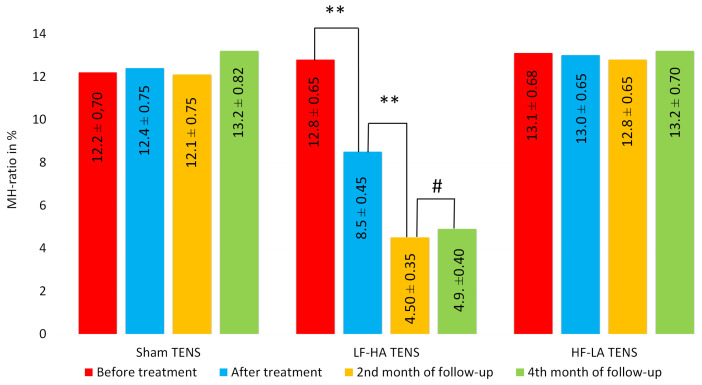
The dynamics of maximal hypoesthesia area in hypoesthesia region after treatment and in the follow-up in the control group and treatment subgroups. Notes: MH-ratio—the area of maximal hypoesthesia divided by the hypoesthesia area in the anterolateral thigh, in %; TENS—transcutaneous electrical nerve stimulation; LF-HA—low-frequency high-amplitude; HF-LA—high-frequency low-amplitude; #—*p* > 1; **—*p* ≤ 0.01.

After treatment, the reduction in the hypoesthesia zone and area was significant only after the use of LF-HA TENS and averaged 26.6% (t = 2.02, *p* = 0.033). A slight decrease in H-ratio was observed with HF-LA TENS, and in some cases (8 limbs) reached 20%. However, the mean H-ratio decreased by only 7.42% (t = 0.586, *p* = 0.562) and was not statistically significant (Figure 10). Likewise, the MH-ratio decreased exclusively after LF-HF TENS, reaching 36.6% (t = 5.43, *p* = 0.0001), and did not change after HF-LA TENS or sham TENS (Figure 11).

At the end of the 2nd month of follow-up, the area of hypoesthesia, assessed by H-ratio, continued to regress after completion of the LF-HA NENS course by another 26.3% (t = 2.05, *p* = 0.047). However, more pronounced changes were found in the central area of maximal hypoesthesia, assessed by HF-ratio, which decreased by another 47.1% (t = 7.017, *p* = 0.0001).

Statistically significant changes at the end of the 4th month of the follow-up period were not registered in any of the examined groups. As a result, the decrease in H-ratio and MH-ratio reached 25.3% (t = 2.03, *p* = 0.048) and 42.4% (t = 10.4, *p* = 0.0001), respectively, compared with the values after treatment and up to 41.2% (t = 4.13, *p* = 0.002) and 61.7% (t = 10.4, *p* = 0.0001) compared with the initial values before treatment. No improvements from baseline in H-ratio and MH-ratio values were recorded in the sham TENS group or the HF-LA TENS group.

### 3.5. Quality of Life Enjoyment and Satisfaction

The quality-of-life enjoyment and satisfaction were evaluated before treatment, at the end of the 2nd and 4th months of follow-up using the Q-LES-Q-SF scale.

**Figure 12:**
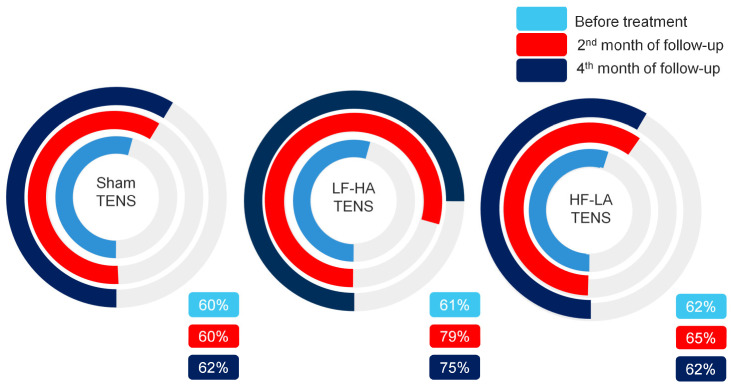
Dynamics of quality of life, determined using quality of life enjoyment and satisfaction scale after treatment and during the follow-up in control group and treatment subgroups. Notes: TENS—transcutaneous electrical nerve stimulation; LF-HA—low-frequency high-amplitude; HF-LA—high-frequency low-amplitude.

The degree of pleasure and satisfaction assessed before treatment was moderately low in all patients and averaged 61 ± 3.5%. No significant differences were recorded between groups (Figure 12).

At the end of the 2nd and 4th months of follow-up, no significant changes were registered in sham TENS and HF-LA TENS groups. Nonetheless, in the LF-HA TENS group, the improvement of quality of live enjoyment and satisfaction significantly increased relative to baseline by 29.5 (t = 2.67, *p* = 0.011) % at the 2nd month follow-up and by 22.9% (t = 2.08, *p* = 0.043) at the 4th month of follow-up.

### 3.6. Electroneuromyography Findings

**Figure 13:**
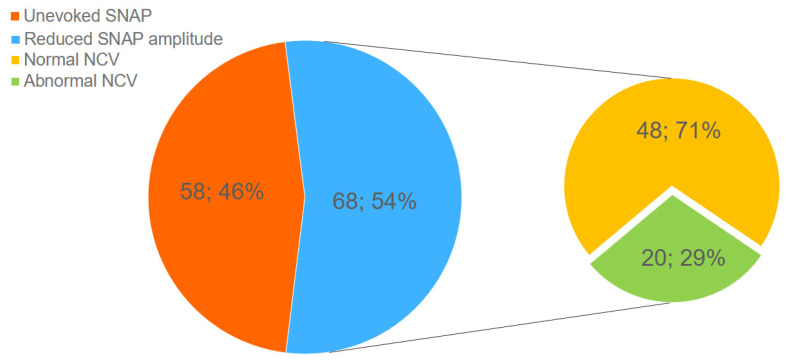
ENMG abnormalities in the studied patients.

Our study included only patients whose ENMG revealed signs of neuropathy of LFCN. Electroneuromyography abnormalities were manifested in a reduction in SNAP amplitude lower than 3 μV. In 46% (*n* = 58) of nerves, SNAP was not evoked due to severe axonopathy. In the remaining nerves (*n* = 68), the average amplitude was 1.1 ± 0.09 μV. A decrease in NCV was recorded only in 29.0% (*n* = 20) of affected nerves in which SNAP could be detected. The decrease in NCV was moderate and averaged 40.9 ± 0.46 m/s (Figure 13).

Before treatment, SNAP amplitude was similar across TENS groups, and the average in the sham tENS group was 0.93 ± 0.14 μV, in the LF-HA TENS group was 1.11 ± 0.17 μV, and in the LF-HA TENS group was 1.30 ± 0.16 μV. Decreases in NCV were detected in six nerves in the sham TENS group, nine nerves in the LF-HA TENS group, and five nerves in the HF-LA TENS group. The mean of NCV in the sham TENS group was 40.8 ± 0.83 m/s, in the LF-HA ENS group—40.4 ± 0.47, and in HF-LA TENS group—41.6 ± 1.02 m/s. No notable differences were detected between groups.

**Figure 14:**
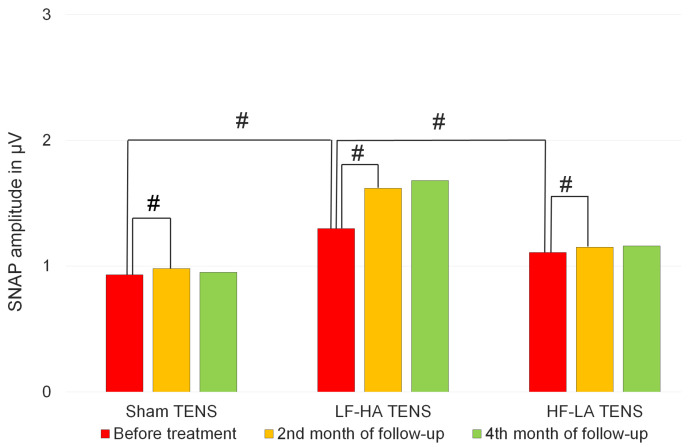
Sensory nerve action potential amplitudes of lateral femoral cutaneous nerve before treatment and during the follow-up in the control group and treatment subgroups. Notes: SNAP—sensory nerve action potential; TENS—transcutaneous electrical nerve stimulation; LF-HA—low-frequency high-amplitude; HF-LA—high-frequency low-amplitude; #—*p* > 1.

At the end of 2nd month of follow-up, no changes were noted in sham TENS and HF-LA TENS groups. In the LF-HA TENS group, a 60% increase in SNAP amplitude was observed in five patients with moderate changes. Overall, the mean SNAP amplitude in this group increased by 24%. However, these changes were not noticeable due to the small sample size. Further, these changes reliably remained unchanged over the next 2 months of follow-up (Figure 14).

### 3.7. Ultrasonography

Before treatment, the following changes were detected in examined nerves at ASIS: nerve abrupt caliber change, indistinct perineurium of the nerve, decrease in intraneural vascularity, and increased cross-sectional area. At the end of the second month of follow-up, no significant changes were registered in sham TENS and HF-LA TENS groups. In the LF-HA TENS group, moderate changes were detected, represented mainly by a decrease in the increased cross-sectional area. However, these changes were not comparable and were not statistically analyzed for comparison with baseline values or between groups.

### 3.8. Correlation Analysis

**Figure 15:**
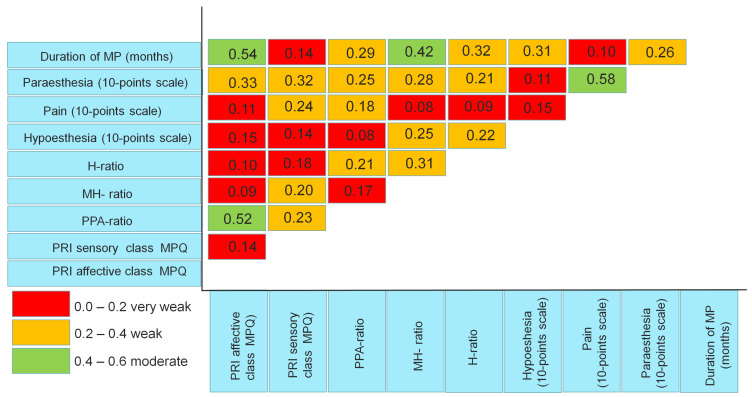
Correlation coefficients between the characteristics of pain, paraesthesia, hypoesthesia, and duration of the disease identified before treatment in all examined patients. Notes: MP—Meralgia paresthetica; H-ratio—hypoesthesia ratio; MH-ratio—maximum hypoesthesia ratio, PPA-ratio—pain and paraesthesia ratio; PRI—pain rating index; MPQ—McGill Pain Questionnaire.

A correlation analysis using Pearson’s correlation coefficient was carried out between the severity and area of pain, paraesthesia, and hypoesthesia in the affected anterolateral thigh and disease duration. The analysis included baseline data identified before treatment (Figure 15).

- Correlation coefficients indicate the strongest relationship (moderate) between disease duration and the severity of pain in affective class of MPQ (r = 0.54, *p* = 0.001), between disease duration and MH-ratio (r = 0.42, *p* = 0.001), between pain and paraesthesia assessed by 10-point scale (r = 0.58, *p* = 0.001), and between PPA—ratio and the severity of pain in affective class of MPQ (r = 0.52, *p* = 0.001).
- A significant weak correlation relationship was found between the severity of pain in the affective class of MPQ and paraesthesia assessed by a 10-point scale (r = 0.33, *p* = 0.005).
- The correlation coefficients between the severity of pain in the sensory class of MPQ, on one side, and paraesthesia assessed by a 10-point scale (r = 0.32, *p* = 0.005), pain assessed by a 10-point scale (r = 0.24, *p* = 0.05), MH-ratio (r = 0.2, *p* = 0.05), and PPA-ratio (r = 0.23, *p* = 0.05), on the other side, were also weak.
- A reliable weak correlation was recorded between the PPA-ratio and variable data of H-ratio (r = 0.21, *p* = 0.05), pain assessed by a 10-point scale (r = 0.20, *p* = 0.05), paraesthesia assessed on a 10-point scale (r = 0.25, *p* = 0.025), and with disease duration (r = 0.29, *p* = 0.025).
- MH-ratio had also weak correlation with H-ratio (r = 0.22, *p* = 0.05) and paraesthesia assessed on a 10-point scale (r = 0.28, *p* = 0.025).
- The relationship between H-ratio and hypoesthesia and paraesthesia, assessed on a 10-point scale, was weak (r = 0.22, *p* = 0.05). However, a stronger association was observed between H-ratio and disease duration (r = 0.32, *p* = 0.001).
- The correlation between the duration of the disease on the one hand and paraesthesia and hypoesthesia, assessed on a 10-point scale on the other, was also weak and amounted to 0.31 and 0.26 (*p* = 0.01), respectively.

## 4. Discussion

To realize the stated purpose of our study, we conducted a comparative analysis between LF-HA TENS, HF-LA TENS, and sham TENS in the treatment of patients with bilateral obesity-related meralgia paresthetica. The patients received 15 sessions of TENS combined with standard pharmacotherapy during the first month. Then, over the next 4 months, a prolonged effect of TENS was observed. In addition to the generally accepted scale methods for assessing pain, paraesthesia, and hypoesthesia, we examined the distribution of pain and paraesthesia by PD with the calculation of PPA-ratio and determined the area of hypoesthesia and maximum hypoesthesia by calculation of H-ratio and MH-ratio.

### 4.1. Correlation Analysis Between Scoring and Area Methods for Assessing Pain, Paraesthesia and Hypoesthesia

It is noteworthy that before treatment, the average severity and area of pain syndrome of paraesthesia and hypoesthesia did not differ significantly between the right and left sides. However, the severity and area of pain, hypoesthesia, and paraesthesia in 87.3% (*n* = 55) patients were unsymmetrical. Correlation analysis revealed that the relationship between the severity of pain and paraesthesia, assessed on a 10-point scale, and the area of location and dispersion of pain and paraesthesia, assessed by PD, was weak but significant. The same result was reported by many authors in assessing pain syndrome of other etiologies [13,36,37,39]. It is important to note that the changes in PRI of an affective class of MPQ are moderately correlated with PPA. Moreover, the correlation coefficient with the intensity of pain in the affective class turned out to be 2 times stronger than with the intensity of pain in the sensory class of MPQ. Additionally, the relationship between the PPA ratio and the disease duration was 2 times stronger than with the level of pain according to VAS. Thus, it has been established that PD is more associated with the affective reactions of patients to long-term chronic MP than with pain intensity. These results are in line with previous evidence obtained in several studies examining the characteristics of PD in other diseases [65,66,67]. Moreover, in many cases, the PPA-ratio exceeded one unit, which indicates that the localization of the pain syndrome exceeds the area of origin or innervation of the nerve. This fact is possible due to the addition of non-neurogenic pain (somatoform) during the chronic course of the disease, which is observed by many authors in patients with other chronic pain syndromes [68,69,70].

In our study, we found that there was a weak correlation between the area of hypoesthesia and the 10-point hypoesthesia score identified during a neurological examination. However, similar results have not been published in the literature. Moreover, we were the first to use the H-ratio to estimate the area of hypoesthesia and the MH-ratio to estimate the fractional distribution of maximum hypoesthesia in the affected anterolateral thigh. Despite the fact that the correlation between the MH-ratio and the H-ratio is 0.32, the MH-ratio was found to be more strongly correlated with disease duration than the H-ratio. We assume that at the onset of the disease, hypoesthesia increases depending on the number of affected nerves, including swollen and inflamed ones. However, the area of maximum (pronounced) hypoesthesia increases as the nerve fibers are damaged (the number of atrophied nerve fibers). Thus, the MH-ratio depends to a greater extent on the disease duration than the H-ratio.

### 4.2. Efficiency of Transcutaneous Electrical Nerve Stimulation in the Treatment of MP

The results obtained leave no doubt that TENS is more effective than sham TENS in the treatment of clinical manifestations of MP. The effectiveness of TENS lies not only in its pronounced analgesic effect, which reduced the severity of pain and paraesthesia symptoms on a 10-point scale but also in reducing the area of their irradiation. The analgesic effect of TENS was demonstrated in the treatment of MP [3,15,16,46], Low back pain [71,72], carpal tunnel syndrome [38,39], neuropathic pain [29,36,37], and pain of various etiologies [73]. In addition to the pain relief, TENS has a recovery effect, expressed in a decrease in the severity of hypoesthesia, determined using scoring methods as well as in a reduction in the area of detected hypoesthesia in H-ratio and MH-ratio. The recovery effect of TENS has been noted in our previous studies in the treatment of MP [2,15], carpal tunnel syndrome [38,39], distal polyneuropathy [36,37,40] and has been demonstrated by other researchers [74,75,76]. It should be noted that the analgesic effect of sham stimulation was moderate and was most likely due to the effects of pharmacotherapy and the psychological effect of placebo [77,78]. However, the regression of intensity and area of hypoesthesia after using sham TENS was negligible.

Many studies have noted a prolonged effect of TENS due to a decrease in the activity of central and peripheral sensitization [36,37,79,80], acceleration of regenerative processes [32,38,39,81], and regression of degenerative changes [38,39,82,83] in the area of application of TENS, as well as in remote lesions [40,84,85] due to the influence of central growth factors [86,87] and the propagation of electrical impulses along the conductive paths [40].

In general, during the first 2 months after the end of treatment, pain and hypoesthesia levels remained without remarkable dynamics. An increase in pain and paraesthesia symptoms was registered between the 2nd and 4th months of observation. However, despite this, the pain syndrome was below the initial level on a 10-point scale by 41.5% and less irradiation by 36.9%. A very important result of our study is the sustained recovery effect after the use of effective TENS over a 4-month follow-up period. However, given the resumption of pain after 2 months of follow-up, we cannot exclude the possibility of deterioration in the function of the LFCN in the long-term follow-up period.

### 4.3. Comparative Analysis Between the Analgesic and Recovery Effect of HF-LA TENS and LF-HA TENS

Several studies have been devoted to comparative analysis between HF-LA TENS and LF-HA TENS in the treatment of many diseases [88]. As far as we know, none of the previous studies have compared the efficiency of different modalities of TENS in the treatment of patients with MP.

#### 4.3.1. Analgesic and Recovery Effect of HF-LA TENS

HF-LA TENS had a more pronounced analgesic effect immediately after treatment. However, the analgesic effect was not persistent and had short, prolonged properties. During the observation period, the pain syndrome gradually returned, and, compared with LF-HA TENS, by the end of the fourth month of follow-up, the analgesic effect was significantly less. In accordance with the findings of many experimental and clinical studies [88,89,90], in our opinion, the benefit of HF-LA TENS during and immediately after treatment is due to segmental analgesic mechanisms directly proportional to the frequency of the applied current. The segmental analgesic mechanism of HF-LA TENS is due to increased afferentation of myelinated sensory fibers that stimulate the gelatinous substance of Rolando, the inhibitory gate of nociceptive afferentation, into the spinal cord [28].

The decrease in the severity and area of hypoesthesia was moderate and reversible within the first 2 months of follow-up. Agreeing with the results of studying the dynamics of hypoesthesia in patients with severe pain, we associate this unstable improvement in sensory function with the decrease in central sensitization-related hyposensitivity of the somatosensory system to non-noxious mechanical stimuli [91,92,93,94]. In a number of studies was reported that this functional hypoesthesia is reversible and regresses after the pain subsides [95,96]. Nevertheless, it is important to note that the reduction in the area of hypoesthesia assessed by H-ratio, but not by MH-ratio, immediately after treatment was 74.5% greater compared with hypoesthesia assessed by scoring methods. This effect indicates that H-ratio includes reversible pain-related nondermatomal somatosensory deficits, which clearly respond to HF-LA TENS pain-relieving therapy.

#### 4.3.2. Analgesic and Recovery Effect of LF-HA TENS

LF-HA TENS differs from HF-LA TENS in its stable analgesic effect, which begins after treatment and intensifies during the first 2 months of observation. In addition to the sensory antinociceptive effect, LF-HA has a distinct inhibitory effect on the emotional-affective aspect of pain, enhancing its analgesic effect. It is this effect that explains the ability of LF-TENS to reduce the area of pain irradiation, and the spread of paresthesia compared with HF-LA immediately after treatment and during the follow-up.

The anti-affective effect of LF-HA TENS has been observed as a secondary outcome in the treatment of many diseases [96,97,98,99,100,101,102]. However, a recent study demonstrated the superiority of LF-HA TENS over HF-LA TENS in reducing the clinical and neurophysiological manifestations of anxiety disorders after stimulation of the right median nerve by an average of 39% [103].

It has been experimentally proven that LF-HA TENS contribute to an increase in the concentration of GABA and activation of GABA(A) receptors in the spinal cord [104,105,106] and brain [107]. In addition to this effect, LF-HA TENS stimulates the release of endogenous central endorphins [108]. In addition to the analgesic effect, LF-HA TENS has an anxiolytic effect, proven clinically and experimentally. The selective effect of LF-HA TENS on central emotion-regulating structures was detected using functional magnetic resonance imaging studies. LF-HA TENS, on the one hand, reduces limbic brain activity with a decrease in excitation of the amygdala, hippocampus, parahippocampal gyrus, and middle and superior temporal gyrus, and on the other hand leads to selective excitation of other central structures, such as the insula, precentral gyrus and thalamus [109,110]. LF-HA TENS is known in the literature as acupuncture-like TENS [111]. In the lateral part of the thigh, 5 cun above the transverse popliteal line, high analgesic and recovery effect acupoints Fengshi (GB-31) and Zhongdu (GB-32) of the gall bladder channel are localized [112]. The action of these points has not only local and segmental effects [113,114] but also generalized ones [115,116]. However, a number of studies have demonstrated the effectiveness of stimulation of these points in the treatment of patients with MP [117,118].

Pain drawings were found to be associated with psychological states in patients with chronic pain [65]. In this regard, in patients with pronounced affective reactions to chronic long-term pain syndrome, pain and paraesthesia symptoms often spread far beyond the affected dermatome [119]. Understanding these mechanisms clarifies the paradoxical phenomenon of the superiority of HF-LA TENS in reducing 10–point scale pain with the simultaneous advantage of LF-HA TENS in reducing the areas of pain irradiation in PD after treatment.

The reduction in the severity of hypoesthesia, noted on a 10-point scale, was 1.44 times superior after LF-HA TENS compared to HF-LA TENS, with a gradual increase in this effect after LF-HA TENS and a decrease after HF-LA TENS during the first 2 months of follow-up, which ultimately resulted in sustained sensory recovery only after LF-HA TENS at follow-up. In six patients, the decrease in h-ratio was greater than the decrease in hypoesthesia determined on a 10-point scale by 1.6 times. These patients had high MPQ affective class scores. However, on average, these scores were 32.7% at the end of the 2nd month of follow-up and 35.5% at the end of the 4th month of follow-up. Positive dynamics of regression of MH-ratio was noted only after LF HA TENS and was higher than hypoesthesia, assessed on a 10-point scale, by 45.9% at the end of 2nd month of follow-up and by 54.3% at the end of the 4th month of follow-up. Growing evidence indicates that the recovery effect of LF-HA TENS is more pronounced with the regression of not only sensory and motor deficits but also with the improvement of the neurophysiological state of the affected nerves [36,37,38,39,40,120,121,122]. Ultimately, despite the positive effect seen immediately after HF-LA, a significant improvement in quality of life was noted only after LF-HA TENS at the end of the 2nd month of follow-up. However, these changes were moderate. In our opinion, the slight improvement is associated with obesity and its consequences, which, despite the relief of pain and sensory deficits, play a decisive role in limiting movement and hindering daily activities.

## 5. Limitations

A very important limitation of our study is the comparison of unimodal TENS using only HF-LA TENS or LF-HA TENS. We believe that a comparative analysis of unimodal and combined use of HF-LA TENS and LF-HA TENS can provide very important results for the clinical use of TENS in the treatment of MP. We suggest that the results obtained may be useful for creating an algorithm for the use of unimodal and combined TENS methods in the treatment of MP, depending on the clinical manifestations of this disease. However, this task was not the goal of our study, and we leave it for future research. Another limitation of our study is the lack of analysis of the effectiveness of TENS in relation to BMI reduction in the treatment of patients with obesity-related MP. Unfortunately, this problem could not be solved due to the short observation period, as well as the lack of a special diet and exercise for the treatment of obesity and overweight. In our study, there was no significant decrease in BMI in the study groups.

## 6. Conclusions

Despite the greatest analgesic effect of HF-LA TENS, assessed by scoring methods, during and after treatment, the reduction in the area of pain and paraesthesia symptoms and the area of hypoesthesia is moderate, short-term, and reversible. In contrast, LF-HA TENS has a pronounced analgesic and sustained anti-paraesthesia effect, manifested by a noticeable decrease in pain and paraesthesia symptoms area in PD, which gradually increase during the first 2 months of follow-up and are accompanied by an irreversible prolonged decrease in the area of hypoesthesia. The area of hypoesthesia correlates with affective reactions to long-term chronic pain, which noticeably regresses under the influence of LF-HA TENS, which, in addition to the analgesic effect, has a pronounced anti-affective effect. The use of pain drawing for assessment of pain and paraesthesia symptoms and body drawing for localization of hypoesthesia area, in combination with scoring methods, significantly increases the accuracy of diagnosis, treatment control, and prognosis of the course of MP. The H-ratio and MH-ratio that we proposed for the first time can help differentiate zones of functional hypoesthesia from organic ones. Considering the safety, simplicity, low cost, minimal side effects, multifactorial mechanisms of pathogenesis, and prolonged effect, it is recommended to use this method in the treatment of patients with MP and various forms of neuropathy.

## Figures and Tables

**Table 1:** Demographic and clinical characteristics of the participants.

	Control Group Sham TENS	Treatment Group		
		HF-LA TENS	LF-HA TENS	*p*
No	22	22	22	*p* > 0.05
Age (years)	48.0 ± 5.45	46.8 ± 5.25	45.2 ± 4.94	*p* > 0.05
Gender (female:male)	10:12	12:10	12:10	*p* > 0.05
Body mass index (kg/m^2^)	34 ± 4.12	35 ± 3.95	35 ± 4.17	*p* > 0.05
Disease duration (months)	15.7 ± 5.20	16.2 ± 4.58	15.8 ± 5.07	*p* > 0.05
Pain by 10-point VAS (points)	6.35 ± 0.19	6.5 ± 0.18	6.5 ± 0.19	*p* > 0.05

Note: TENS—Transcutaneous electrical nerve stimulation; LF-HA—low-frequency high-amplitude; HF-LA—high-frequency low-amplitude; VAS—visual analog scale; *p*—level of marginal significance, Mean ± SEM.

**Table 2:** Characteristics of TENS pulses.

	Frequency	Duration	Amplitude
Sham TENS	1 Hz	50 µs	Sensory threshold
LF-HA TENS	1 Hz	200 µs	10 mA above the motor threshold of underlying muscles
HF-LA TENS	50 Hz	50 µs	5 mA above sensory threshold

Note: TENS: transcutaneous electrical nerve stimulation; LF-HA: low-frequency high-amplitude; HF-LA: high-frequency low-amplitude; Hz: Hertz; µs: microsecond; mA: milliampere.

**Table 3:** PRI of MPQ in sensory and affective classes before and after treatment of patients with MP by TENS.

Sensory Class	Before Treatment	After Treatment	Follow-Up Period (2 Months)	Follow-Up Period (4 Months)
Sham TENS	15.1 ± 0.48	13.1 ± 0.60 *	12.9 ± 0.60 *	13.2 ± 0.62 *
LF-HA TENS	15.4 ± 0.48	8.2 ± 0.32 *#	5.5 ± 0.34 *#	5.8 ± 0.30 *#
HF-LA TENS	15.2 ± 0.47	5.9 ± 0.25 *#	8.0 ± 0.32 *#	8.2 ± 0.35 *#
**Affective Class**	**Before** **Treatment**	**After** **Treatment**	**Follow-Up Period** **(2 Months)**	**Follow-Up Period** **(4 Months)**
Sham TENS	8.3 ± 0.25	8.4 ± 0.25 *	8.4 ± 0.25 *	8.5 ± 0.24 *
LF-HA TENS	8.1 ± 0.26	3.2 ± 0.15 *#	2.4 ± 0.13 *#	2.7 ± 0.14 *#
HF-LA TENS	8.3 ± 0.26	5.5 ± 0.19 *#	6.5 ± 0.16 *#	6.4 ± 0.17 *#

Note: TENS—transcutaneous electrical nerve stimulation; LF-HA—low-frequency high-amplitude; HF-LF—high-frequency low-amplitude. *—*p*-value ≤ 0.05—statistical significance between initial values and results after treatment; #—*p*-value ≤ 0.05—statistical significance between treatment subgroups and control group.

## Data Availability

The original contributions presented in this study are included in the article. Further inquiries can be directed to the corresponding authors.
